# The Influence of Selected Psychological Factors on Medication Adherence in Patients with Chronic Diseases

**DOI:** 10.3390/healthcare10030426

**Published:** 2022-02-24

**Authors:** Monika Bąk-Sosnowska, Magdalena Gruszczyńska, Julia Wyszomirska, Anna Daniel-Sielańczyk

**Affiliations:** Department of Psychology, Chair of Social Sciences and Humanities, School of Health Sciences in Katowice, Medical University of Silesia in Katowice, 40-752 Katowice, Poland; monika.bak-sosnowska@sum.edu.pl (M.B.-S.); mgruszczynska@sum.edu.pl (M.G.); jwyszomirska@sum.edu.pl (J.W.)

**Keywords:** adherence, health locus of control, mindfulness, chronic disease, psychological factors, stress coping

## Abstract

Background: Insufficient adherence to treatment is a relevant problem. This study aims to determine the impact of health locus of control, stress coping style and level of mindfulness on medication adherence in patients with a chronic illness. Methods: The study included 768 people. The diagnostic survey involved the use of: Medication Adherence Questionnaire (MAQ), Multidimensional Health Locus of Control Scale (MHLC), The Coping Inventory for Stressful Situations (CISS), and The Mindful Attention Awareness Scale (MAAS). Results: Participants were divided into two subgroups, i.e., adherent (*n* = 219) and non-adherent (*n* = 549). We observed significant differences between the subgroups in age, BMI, sex, place of residence, education, and for all MHLC subscales, two CISS subscales and MAAS. The identified medication adherence variables were: female gender (OR = 1.55), BMI (OR = 0.95), MHLC/Internal (OR = 0.95), CISS/Emotional (OR = 1.03), MAAS (OR = 0.97). Conclusions: A strong internal health locus of control, a higher level of mindfulness and a lower level of emotional-stress coping style increase the likelihood of adherence with medication recommendations in patients with chronic diseases.

## 1. Introduction

Chronic non-communicable diseases (NCDs), such as cardiovascular diseases, cancer, diabetes, and respiratory diseases, are associated with a higher morbidity and mortality than all other causes combined. Estimates suggest that, by 2030, the average global mortality due to NCDs will be 75.26% [1]. The aforementioned diseases also cause a major social and economic burden and comprise a substantial cause of disability, especially in the female population [2,3]. The vast majority of chronically ill people living in highly developed countries remain in the health care system under long-term observation, supervision or medical care. They receive assistance in diagnosis and appropriate treatment [4]. An inherent element of treatment is medical recommendations regarding pharmacotherapy and, often, lifestyle. Patient adherence has a direct impact on the effectiveness of treatment [5]. 

Adherence means the extent to which a patient’s behavior conforms to the recommendations of their physician. It comprises a behavioral continuum, ranging from complete refusal to take prescribed medication (completely non-adherent behavior), through partial adherence to treatment regimens (partially adherent behavior), to precise and regular medication use (fully adherent behavior) [6]. The unfortunate estimate is that approx. 50% or more patients undergoing long-term treatment for somatic disorders either do not take their medications in a proper manner or cease taking them altogether [7]. A Polish study conducted on a population of over 63.000 chronically ill people demonstrated that as many as 83.8% of respondents failed to comply with medical advice in various ways [8]. 

Non-adherence with medication recommendations is manifested, among others, by failure to purchase the prescribed medication, failing to start taking the prescribed medication, skipping one’s medication, and self-adjustment of the dose, frequency of dose or duration of treatment. This will result in disease progression, lower patient quality of life, increased use of healthcare resources, and increased morbidity and mortality [9]. Non-adherence is poorly explained by the traditional medical model, which assumes that a medical recommendation based on scientific evidence is best for the patient, and that the proven efficacy thereof is a sufficient recommendation to encourage the patient to comply. Furthermore, it assumes that the only possible obstacle may be socioeconomic factors (e.g., gender, age, race and education) [10]. This model does not provide for the patient’s subjective perspective and the influence of disease-related factors (e.g., knowledge about the causes of the disease, disease course and treatment), the recommended treatment (e.g., views on taking medication, perceived effectiveness and side effects, treatment costs, treatment acceptability, access to treatment, doctor–patient relationship), or life situation (e.g., life circumstances, available resources, including social support, and competitive priorities) [11,12,13,14].

Health-related decision-making is also influenced by psychological factors. In this study, particular attention was paid to subjective psychological factors, whose relationship with adherence has not yet been widely explored. The authors were guided in their choice by the fact that psychological factors were connected with health, as well as their varying degrees of stability and modifiability. This may have significant practical implications related to the selection of adequate interventions, enhancing adherence. 

Health Locus of Control (HLC) is relatively stable and refers to beliefs concerning the source of influence on one’s own health. It can be located inside the person (Internal HLC) or outside of it (External HLC), and based in authority (Powerful Others HLC) or in chance (Chance HLC) [15,16]. Health Locus of Control is an indicator used to test patients’ health-related beliefs. Studies show that HLC is an independent predictor of medication adherence, which is better in people with external HLC and weaker in people with internal and chance HLC [17,18]. Furthermore, it is associated with self-care and a healthy lifestyle, which are very important in NCDs [19]. 

The style of coping with stress, the most stable factor, is understood as a combination of cognitive and behavioral efforts to control, reduce or eliminate stress. The task-oriented style is associated with focusing on the problem and actively seeking information and solutions. Going further, the emotional style reflects attempts to reduce stress through emotional responses (e.g., anger, blame) or rumination. Finally, the avoidance style refers to distraction and withdrawal in order to avoid stress, which may take the form of substitute activities or excessive social involvement [20]. Chronic disease is a stress stimulus; therefore, the effectiveness of adaptation to a disease, including adherence to medication advice, depends, inter alia, on the individual’s appropriate coping strategy. For example, it was found that adherent versus non-adherent patients used more active coping strategies (i.e., seeking information about illness and therapy), and also reported more diverting (i.e., distracting with something pleasant) and self-encouraging (i.e., seeking success, indulge oneself) behavior [21]. The results of a study conducted in a group of oncological patients confirmed a significant correlation between the level of medication adherence and the use of avoidance-oriented strategies of coping with stress, including willingness to engage in social relationships [22]. Additionally, a study in a group of patients with rheumatological diseases showed that a low level of internal health locus of control increased the likelihood of medication adherence [23]. 

Mindfulness, the most modifiable factor, related to awareness of emotions, needs and the ability to interpret signals from the body, represents a somewhat purposeful, non-judgmental attention to the present moment. This may be an important factor in the process of treatment and medication adherence [24]. Its relationship with health, including adherence with medication advice, has been confirmed in patients with both somatic [25] and mental illness [26]. The conceptual model created by Salmoirago-Blotche and Carey assumes that mindfulness training could improve adherence via improvements in attention and working memory, sleep, stress, and depressive symptoms. Positive changes in adherence will, in turn, result in improvements in biomarkers and clinical outcomes. We posit that mindfulness training could improve adherence via improvements in attention and working memory, sleep, stress, and depressive symptoms. Positive changes in adherence will, in turn, result in improvements in biomarkers and clinical outcomes [27]. Research reviews show that mindfulness training has a great potential in supporting patient cooperation in the treatment process. Many studies confirm the positive effect of mindfulness on medication adherence. At the same time, the frequently used form of self-reporting on medication adherence, and the high risk of bias in the conducted research, do not allow for an unambiguous assessment of this relationship [28]. This is a compelling reason to continue research into the relationship between mindfulness and medication adherence. 

Even though studies that analyze chronically ill people’s adherence to medication advice also consider psychological aspects, they often focus on a concrete age group [29,30] or patients affected by a specific disease [31,32]. Therefore, there is a need to determine the extent to which psychological factors with a confirmed relationship to health-related behaviors influence medication adherence in chronically ill people. 

The health locus of control and style of coping with stress are relatively stable constructs; therefore, it is important that physicians take them into account in direct contact with the patient and select appropriate interventions when issuing recommendations and cooperating with the patient. Mindfulness is a psychological process that can be developed through exercise or meditation. Determining the impact of the variables selected in this study on medication adherence could be useful for predicting the level of patient cooperation in the treatment process and for creating a psychological support system supporting the treatment of chronic diseases. 

The study aimed to determine the health locus of control, style of coping with stress, and the level of mindfulness regarding adherence to medication advice by chronically ill people.

## 2. Methods and Participants

Patients diagnosed with a chronic disease remain in contact with the physician and participate in regular medical consultations. During follow-up visits, the patient’s health condition, treatment effectiveness, medication adherence are analyzed and, if necessary, further recommendations are issued. 

The study included people who met the following inclusion criteria: age of at least 18, at least 6 months from diagnosis of a chronic disease, having medical advice for pharmacotherapy for a chronic disease, psychophysical condition allowing participation in the study. The study was anonymous; furthermore, learning the purpose of the study and voluntarily returning the completed questionnaires was tantamount to providing informed consent to participate.

The exclusion criteria were: dementia; mild cognitive impairment; and mental disorders that prevent controlling medication alone, such as a current major depressive episode or psychotic disorder.

We distributed 1000 questionnaires, of which 843 were returned. The exclusion criteria were considered and revealed a pool of 790 respondents After the rejection of incomplete questionnaires, 768 people of average age 57.11 ± 15.77, including 520 females and 248 males, were finally enrolled.

### 2.1. Method

The study was cross-sectional. We used the diagnostic survey method with four standardized questionnaires. We also employed a self-questionnaire, which included socio-demographic questions (gender, age, body weight, height, education, place of residence, partner status, having children, professional status, attitude to faith) and health-related questions (chronic diseases, having received medication advice).

Adherence with medication advice was assessed using a Polish adaptation of The Medication Adherence Questionnaire (MAQ) [33]. The questionnaire consists of four dichotomous questions, which relate to forgetfulness and reasons for failing to take prescribed medication. The respondent scores 0 points for each affirmative answer and 1 point for each negative answer. The final score range is between 0 and 4 points. For research purposes, low (0–1), medium (2–3) and high (4) adherence levels can be distinguished [34]. The internal consistency of the questionnaire was estimated at Cronbach’s alpha = 0.63.

Health locus of control was assessed using the Polish adaptation of the Multidimensional Health Locus of Control Scale (MHLC) [35]. The scale contains 18 statements and includes beliefs about general expectations in three areas of health locus of control: internal (IC), the powerful others (PO), chance (Ch). The respondent expresses their attitude towards the presented statements (where 1—totally agree, 2—agree, 3—rather agree, 4—rather disagree, 5—disagree, 6—totally disagree). The higher the score, the stronger the belief that a given factor affects one’s health. The internal consistency of the questionnaire varies within the range 0.601–0.706 using Cronbach’s alpha, depending on the subscale. 

To identify a stress coping strategy we used the Polish adaptation of The Coping Inventory for Stressful Situations (CISS) [36]. The CISS consists of 48 items. The respondent refers to each statement specifying the frequency of a reaction (where 1—ever, 2—very rarely, 3—sometimes, 4—often and 5—very often). The results are grouped into three subscales: task-oriented style (TOS), emotional-oriented style (EOS), avoidance-oriented style (AOS). The last subscale is additionally divided into two more: Distraction Style (DS) and Social Diversion Style (SDS). For TOS and EOS, Cronbach’s alpha coefficient values were obtained in the range of 0.82–0.83, while for AOS, the values ranged from 0.61 to 0.71.

The Polish adaptation of The Mindful Attention Awareness Scale (MAAS) was used to determine the level of mindfulness [37]. The scale examines the disposition towards mindfulness. It contains 15 items. The task of the respondent is to assess the frequency of experiencing these items, where 1 means almost always, 2—very often, 3—quite often, 4—rarely, 5—very rarely, 6—almost never. The higher the score, the more mindful the respondent is. The Cronbach’s alpha of the scale was within the range 0.8–0.85.

### 2.2. Study Organization

The study was designed as a cross-sectional study, aimed at assessing the importance of selected psychological factors. It was conducted in five randomly selected primary healthcare centers in Silesia in Poland. Cluster sampling was applied to the test group. Participation in the study was offered to every adult chronically ill patient who registered for a medical visit in the selected GP clinics during the period from January to June 2019. Potential participants were informed about the purpose of the study, its anonymity, voluntariness and the potential to refuse study participation. Respondents completed questionnaires during their stay in the primary healthcare center, while waiting for the visit. The response time to the questions was not limited. Participants returned the completed questionnaires to a secured box specially designated for this purpose. Submitting a completed questionnaires was tantamount to giving informed consent to participate in the study.

### 2.3. Ethics

The research protocol was approved by the local Ethics Committee of the Medical University of Silesia in Katowice and qualified as not being a medical experiment, approval no. KNW/0022/KB/170/17. All clinical investigation was conducted according to the principles expressed in the Declaration of Helsinki.

### 2.4. Statistical Analysis

Statistical analyses were performed using the STATISTICA 14.0 software. The Kolmogorov–Smirnov test was used to assess the normality of distributions. Non-parametric analyses (chi-2 Pearson, U-Mann–Whitney) were used to evaluate the differences between the groups. Linear regression analysis was performed to summarize the analysis. Statistical significance was assessed at *p* = 0.05.

## 3. Results

**Figure 1:**
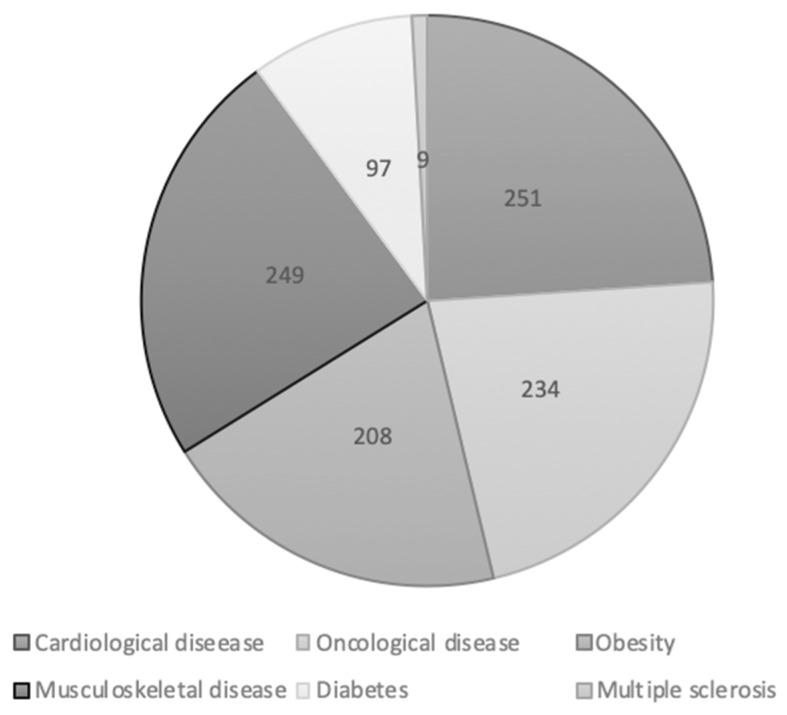
Type of dominant diseases in study group (*n* = 768).

The most common chronic conditions in the study group were cardiological disease, oncological disease, musculoskeletal disease, and obesity. Diabetes and multiple sclerosis were present to a lesser extent. Many people were affected by multiple diseases, but they were asked to indicate the dominant disease for which they had been prescribed pharmacotherapy (Figure 1).

The results of the MAQ questionnaire were used as the basis for dividing participants into two subgroups depending on adherence with medication advice. The Adherent Group (people who have a high and average adherence to medical advice) comprised 28.5% of the respondents (*n* = 219), and the Non-Adherent Group (people who have a low adherence to medication recommendations) comprised 71.5% (*n* = 549). As regards anthropometric data, the subgroups differed significantly in terms of age and BMI (Table 1). 

As regards sociodemographic data, the subgroups differed significantly in sex, place of residence, and education level (Table 2).

There were also major differences between the subgroups regarding all three types of health locus of control, the level of mindfulness, and the task-oriented and emotional styles of coping with stress (Table 3).

The results of multivariate analysis demonstrated that males had a 1.5 times higher risk of non-adherence than females. Participants with a higher BMI parameter exhibited more frequent adherence with medical advice. As the BMI value increased by 1 kg/m^2^, the risk of non-adherence decreased by 1.05 times. The risk of non-adherence also decreased by 1.04 times with an individual increase in the MHLC questionnaire score in the subscale of internal control. On the other hand, a one-point increase in the CISS questionnaire on the emotional orientation subscale translated into a 1.03 higher risk of non-adherence with medical advice. A one-point higher score on the MAAS questionnaire scale decreased the considered risk of non-adherence by 1.03 times (Table 4). 

## 4. Discussion

Medical adherence is extremely important for successful treatment and rehabilitation, which has a direct impact on the patient’s health and quality of life. 

Recent scientific reports indicate that regular medical adherence is challenging for a large proportion of patients [38,39,40]. The aim of our study was to determine whether psychological aspects such as the health locus of control, style of coping with stress and the level of mindfulness affect medication adherence in chronically ill patients.

We demonstrated that a higher medication adherence was related to gender, education and place of residence, as well as BMI. Females exhibited a greater medication adherence than males, as did patients with secondary and higher school education compared to individuals with basic schooling. 

This is confirmed by the literature review carried out by Kardas et al. [41] for 2000–2009, which revealed that females and individuals with higher education follow medical advice more often and that females generally exhibit more pro-health behaviors, e.g., they undergo preventive examinations more often than males. Our study demonstrated that individuals who adhered to medication advice had a slightly higher body weight and BMI than those who did not. This is inconsistent with reports by other authors, in which it was demonstrated, for example, that females suffering from obesity are less likely to follow their doctors’ recommendations for screening for breast and cervical cancer [42]. At the same time, patients with a higher BMI report less readiness to comply with general health advice than patients with a lower BMI [43]. However, regarding the above data, it should be remembered that, in our study, the average BMI in both subgroups (adherent and non-adherent) indicated only slightly overweight, and not obesity. It can be assumed that, along with a noticeably increased BMI, the respondents sought to take better care of their own health, including regular pharmacotherapy. It is also worth noting that in the group of people over 60 years of age, being moderately overweight is an indicator of healthy aging and may at least partially protect against comorbidities [44].

Furthermore, we demonstrated that urban dwellers followed their medication recommendations to a greater extent than those living in the countryside. A large city is often associated with greater availability of medical and multi-specialist care compared to the countryside. Research by other authors confirms that patients report problems with access to and organization of medical services as one of the reasons for medical non-adherence [45]. The above factors can explain the obtained results and, at the same time, should act as a driving force, increasing one’s efforts to make research more accessible and increase the number of medical specialists in smaller towns. It also seems important to be involved in actions promoting health prophylaxis and the patient’s co-responsibility for treatment. 

A major factor in medical adherence is one’s own approach to health and a conviction that the treatment is, in fact, justified. Respecting medical advice is closely related to the health locus of control. Our research showed that people with higher levels of both internal and external health loci of control had greater adherence than people with a chance health locus of control. The higher the respondents’ health locus of control, the lower the risk of medical non-adherence. Similar conclusions were drawn by Cottrell et al. and Percival et al., who conducted research among cardiovascular patients. According to their results, acceptance of disease, positive attitude to the treatment and the belief that treatment brings health benefits all contribute to medical adherence [46,47]. The relationship between inner locus of control and medication adherence is supported by the self-efficacy theory [48]. Self-efficacy is the belief in your own ability to organize and control your behavior on purpose lead to the specified result, expected by yourself as the consequence of this behavior. Nafradi et al. prouved that high levels of self-efficacy and internal health locus of control are consistently found to promote medication adherence [49]. External control dimensions were found to have mainly negative (Chance- and God-attributed control beliefs) or ambiguous (Powerful-Others-attributed control beliefs) links to adherence, except for Doctor Health Locus of Control, which had a positive association with medication adherence. In our study, adherent patient versus non-adherent also had a significantly higher level of health-related locus of control in both the Internal and Powerful others dimensions.

People who are more aware of the importance of regular adherence to medical advice for improving their health have a high appreciation for it [22]. Knowing one’s own body, paying attention to whether it is in good condition, consciously relaxing and paying attention to potentially disturbing symptoms, is characteristic of people with a higher level of mindfulness. Vélez-Vélez and Bosch suggest that the awareness and knowledge of one’s own disease, symptoms, consequences, and treatment methods favor medical adherence [50]. In our research, medication adherence was closely related to higher mindfulness. Other authors reached similar conclusions, and indicated a link between greater disease acceptance, high levels of medical adherence, and mindfulness [26,27,51,52,53]. The role of self-awareness and other psychosocial factors in the treatment process is a key aspect of the biopsychosocial model of health [54]. According to its assumptions, not only biological factors, but also psychological, emotional and social factors have an impact on human health and their attitude in the treatment process. Consequently, the relationship between the patient and the attending physician also plays an important role, which may explain the previously discussed relationship between Powerful Others health-related locus of control and medication adherence.

The results are also reflected in the style of coping with stress, which is important in disease. It refers to characteristic strategies, or ways of coping with difficult situations. As chronic diseases are long-term in nature, how a person deals with the symptoms, as well as the effects of the disease, are of great importance in the adaptation and acceptance of one’s condition. In the studied group of chronically ill patients, individuals with high medical adherence more often had a task-oriented style of coping with stressful situations, while non-adherent patients more often exhibited the emotional style. Many other studies also indicate a link between the task-oriented coping and a higher degree of adherence to medication recommendations [50,55]. This relationship is consistent with the cognitive-transactional theory of stress, which defines coping with stress as the cognitive and behavioral efforts of an individual to deal with requirements that are assessed as overburdening or exceeding one’s resources [56]. However, the style of coping with stress in relation to chronic diseases may be related to the type of disease. A study conducted by Gruszczyńska et al. on a group of oncological patients showed that the respondents who followed medical recommendations to a great extent had an emotional style of coping with stress [22]. Similar results were obtained by Sampaio et al. [55]. Therefore, it can be hypothesized that it is not the mere fact of being affected by a chronic disease, but the disease type, that will trigger specific strategies available in a given coping style. However, this hypothesis requires further research.

Although our study showed the importance of certain psychological variables for medical adherence, it is worth noting that the strength of the influence of these variables was not high (OR from 0.95 to 1.5). This may suggest that these variables should be considered as a second priority when estimating the likelihood of patient adherence. In the first place, we must consider psychological variables with a documented greater influence, such as emotional support (OR 1.83), unidimensional social support (OR 2.35), family cohesiveness (OR 3.03), being married (OR 1.27), and living with someone (for adults, OR 1.38) [57].

### 4.1. Clinical Practice

The psychological factors studied by us (health locus of control, style of coping with stress, level of mindfulness) are, to some extent, modifiable. Therefore, diagnosis of these factors in patients and the further application of targeted psychological interventions may develop these factors in the desired direction, thereby improving medical adherence and, consequently, increasing the effectiveness of chronic disease treatment. The diagnosis and modification of psychological variables falls within the area of health psychology and clinical psychology. Specialists in these fields should be members of the therapeutic team providing care for chronically ill patients. They can not only support the personal development of patients, but also provide guidance for doctors, e.g., on effective patient communication in order to strengthen the desired psychological features in treatment and recovery.

In the light of our own results, we can also formulate some suggestions for a physician caring for chronically ill patients and for managers of health centers. Considering the relationship between inner health-related locus of control and medication adherence, the doctor can strengthen the commitment, responsibility, and self-efficacy of the patient, as well as his involvement in the treatment process and possible modification of pharmacotherapy. The physician should also talk to patients about stress and give them basic advice on how to deal with it better. In addition, it may encourage patients to be more self-aware in the area of the body (self-observation, attention to one’s needs, reactions to drugs), and also encourage the use of behavioral methods that have a positive impact on the regularity of medication (e.g., drug reminder applications, drug dispensers).

Managers of health centers can support the organization of support groups and group training, such as mindfulness or stress management. Many studies support the effectiveness of such interventions in improving medication adherence [28,58].

### 4.2. Scope for Future Research

Based on the results of our own research and on the reports of other authors, we see the need to develop research on an attitudinal symmetry between the doctor and patient, influencing the patient’s control over disease management, including medication adherence.

Additionally, we see a need for researchers to provide strong evidence for the impact of psychological interventions on patient medication adherence. Researchers should prioritize rigorous experimental designs, theory-driven investigations of behavioral mechanisms, and the use of objective measurements of adherence.

### 4.3. Limitations

Our study is characterized by several limitations that should be considered when interpreting the results. First of all, the clinical variables were controlled poorly, since we did not check the actual chronic diseases of patients in their medical records. We merely asked patients to indicate the dominant disease for which pharmacotherapy had been prescribed. This was due to the assumption of study anonymity and a conviction that the subjective perception of the patient’s health and disease is more important than the objective state in the study of psychological factors. While we are aware that mental disorders are common in the population and probably affect some of participants in our study, nobody reported a mental disorder as a dominant chronic disease. For this reason, mental disorders were not included in our study as one of the chronic diseases that have affected our respondents and/or for which they have been prescribed medications. Another limitation is the poor control of the medication advice received by the respondents in relation to their health condition. We did not ask detailed questions about the medications taken, frequency or duration of medication, or whether the respondents have other medical recommendations other than pharmacological ones. The significant advantage of female participants in the study may be seen as a study limitation. Although some diseases included in the study are less common in females than in males, e.g., CVD, data from the Central Statistical Office in Poland confirm that there are more women than men who use outpatient care [59,60].

## 5. Conclusions

A strong internal health locus of control, a higher level of mindfulness and a lower level of emotional-stress coping style increase the likelihood of adherence with medication recommendations in patients with chronic diseases.

## Figures and Tables

**Table 1:** Comparison of anthropometric data between adherent and non-adherent respondents.

Variable	Me (IQR)			*p* *
	Total (*n* = 768)	Adherence (*n* = 219)	Non-Adherence (*n* = 549)	
Age (yrs)	61 (46–69)	64 (53–69)	59 (44–68)	0.002
High (m)	1.65 (1.6–1.73)	1.64 (1.6–1.72)	1.66 (1.6–1.74)	0.068
Weight (kg)	74 (63–86)	76 (63.5–87)	73 (62.75–86)	0.171
BMI (kg/m^2^)	26.29 (23.39–30.11)	27.01 (24.1–30.67)	26.1 (22.93–29.79)	0.003

Me—median; IQR—interquartile range; *p*-value of the level of statistical significance; * U Mann-Whitney test.

**Table 2:** Comparison of sociodemographic data between adherent and non-adherent patients.

Variable		Total (*n* = 768)	Adherence (*n* = 219)	Non-Adherence (*n* = 549)	*p* *
Sex	Female	67.71% (*n* = 520)	74.43% (*n* = 163)	65.03% (*n* = 357)	0.01
	Male	32.29% (*n* = 248)	25.57% (*n* = 56)	34.97% (*n* = 192)	
Place of residence	Rural	18.75% (*n* = 144)	13.24% (*n* = 29)	20.95% (*n* = 115)	0.01
	Urban	81.25% (*n* = 624	86.76% (*n* = 190)	79.05% (*n* = 434)	
Education	Primary school	4.95% (*n* = 38)	4.11% (*n* = 9)	5.28% (*n* = 29)	0.01
	Vocational school	21.61% (*n* = 166)	15.07% (*n* = 33)	24.23% (*n* = 133)	
	Secondary school	41.54% (*n* = 319)	48.4% (*n* = 106)	38.8% (*n* = 213)	
	Higher education	31.9% (*n* = 245)	32.42% (*n* = 71)	31.69% (*n* = 174)	
Employment	In work	35.55% (*n* = 273)	30.14% (*n* = 66)	37.7% (*n* = 207)	0.05
	Out of work	64.45% (*n* = 495)	69.86% (*n* = 153)	62.3% (*n* = 342)	
Partner status	Single	37.37% (*n* = 287)	37.9% (*n* = 83)	37.16% (*n* = 204)	0.91
	In relationship	62.63% (*n* = 481)	62.1% (*n* = 136)	62.84% (*n* = 345)	
Having children	Yes	80.73% (*n* = 620)	81.74% (*n* = 179)	80.33% (*n* = 441)	0.71
	No	19.27% (*n* = 148)	18.26% (*n* = 40)	19.67% (*n* = 108)	
Faith	Believer	91.54% (*n* = 703)	90.87% (*n* = 199)	91.8% (*n* = 504)	0.78
	Non-believer	8.46% (*n* = 65)	9.13% (*n* = 20)	8.2% (*n* = 45)	

*p*-value of the level of statistical significance; * chi square test.

**Table 3:** Comparison of the questionnaire results between adherent and non-adherent patients.

Variable		Me (IQR)			*p* *
		Total (*n* = 968)	Adherence (*n* = 219)	Non-Adherence (*n* = 549)	
MHLC	Internal control	24 (21–27)	25 (22–29)	24 (20–27)	<0.001
	Powerful others	23 (21–26)	24 (21–27)	23 (21–26)	0.0454
	Chance	22 (18–26)	21 (17–25)	22 (18–27)	0.0088
CISS	Task orientated	53 (48–59)	55 (49–60)	52 (47–59)	0.0224
	Emotional orientated	44 (37–50)	42 (35–48)	45 (38–51)	<0.001
	Distraction	21 (17–25)	20 (17–24)	21 (18–25)	0.0513
	Social diversion	16 (14–19)	17 (13–19)	16 (14–19)	0.4837
	Avoidance orientated	46 (40–51)	45 (39–51)	46 (40–51)	0.188
MAAS	Mindful attention	60 (51–70)	65 (56.5–74.5)	58 (49–68)	<0.001

Me—median; IQR—interquartile range; *p*-value of the level of statistical significance; * U Mann-Whitney test.

**Table 4:** Logistic regression model explaining the risk of non-adherence with medication recommendations.

	OR	2.5%	97.5%	*p*
(constant)	42.45	7.819	242.6	<0.001
Sex: male	1.554	1.077	2.264	0.02
BMI	0.95	0.917	0.984	0.004
Internal control	0.958	0.925	0.991	0.014
Emotion orientation	1.031	1.012	1.051	0.001
Mindful attention	0.97	0.956	0.984	<0.001

OR—odds ratio; *p*-value of the level of statistical significance.

## Data Availability

The data presented in this study are available on request from the corresponding author.
